# LXtoo: an integrated live Linux distribution for the bioinformatics community

**DOI:** 10.1186/1756-0500-5-360

**Published:** 2012-07-19

**Authors:** Guangchuang Yu, Li-Gen Wang, Xiao-Hua Meng, Qing-Yu He

**Affiliations:** 1 Institute of Life and Health Engineering and Key Laboratory of Functional Protein Research of Guangdong Higher Education Institutes, Jinan University, Guangzhou, 510632, China; 2 Guangdong Information Center, Guangzhou, 510632, China; 3 College of Information Science and Technology, Jinan University, Guangzhou, 510632, China

**Keywords:** Bioinformatics, Software, Linux, Operating system

## Abstract

**Background:**

Recent advances in high-throughput technologies dramatically increase biological data generation. However, many research groups lack computing facilities and specialists. This is an obstacle that remains to be addressed. Here, we present a Linux distribution, LXtoo, to provide a flexible computing platform for bioinformatics analysis.

**Findings:**

Unlike most of the existing live Linux distributions for bioinformatics limiting their usage to sequence analysis and protein structure prediction, LXtoo incorporates a comprehensive collection of bioinformatics software, including data mining tools for microarray and proteomics, protein-protein interaction analysis, and computationally complex tasks like molecular dynamics. Moreover, most of the programs have been configured and optimized for high performance computing.

**Conclusions:**

LXtoo aims to provide well-supported computing environment tailored for bioinformatics research, reducing duplication of efforts in building computing infrastructure. LXtoo is distributed as a Live DVD and freely available at http://bioinformatics.jnu.edu.cn/LXtoo.

## Findings

The completion of the human genome project [1] fuelled the spark of many post-omics technologies such as massively parallel sequencing, genotyping and proteomics. These technologies dramatically increase biological data generation, and the biological data throughput has been increasing at a rate exceeding Moore’s Law [2]. Along with this revolution comes the challenge of inventing solutions for fast conversion of raw data into biological knowledge. Many talented biologists and programmers have been implementing various methods to address this challenge. While the availability of these methods accelerates biological research, the broad range of the current software packages forces biologists to spend growing amounts of time installing, configuring and maintaining software rather than focusing on research [3]. Moreover, many research groups lack such bioinformatics expertise, and thus often face data analysis bottleneck, leading to the slow progress of biological research.

Building tailored computing solutions requires specialized knowledge. While this challenge can be overcome by using free and open source software (FOSS) [4], the processes of compiling, installing, and configuring software are repetitive, error-prone, time consuming, and sometimes frustrating. Expert system administrators with extensive bioinformatics expertise are rare [4]. As bioinformatics pipelines become more complex and sophisticated, customization of computing solutions also becomes more complex. These problems are compounded by the growth of the bioinformatics field that has produced thousands of tools and web services over the last decade. Therefore, it is desirable to deliver an ideal computer system to the research community to address these issues. This goal can be accomplished by distributing a Linux-based operating system, gearing toward performing bioinformatics analyses. Existing Linux distributions such as BioKnoppix and Quantian have not been updated for years. Some distributions aimed at a specific usership, such as Vigyaan for computational chemistry, BioconductorBuntu [5] for microarray analysis, and phyLIs [3] for phylogenetic analysis; others like Vlinux and DNALinux [6] mostly focused on sequence analysis and protein structural prediction.

Here, we present an integrated live Linux distribution, LXtoo, to provide a comprehensive collection of biological software, including tools for sequence and structural analysis, microRNA target prediction, microarray and proteomics data mining, protein network analyses and even for computationally complex processes like molecular dynamics and modeling.

## Implementation

Developed using FOSS technologies, LXtoo is a freely available Linux distribution based on Gentoo Linux. LXtoo aims to present a fast, flexible, portable, and powerful computing platform, integrating most commonly used bioinformatics tools with pre-compiled, installed, and configured software. LXtoo contains several libraries for high performance computing, including Fastest Fourier Transform in the West (FFTW) for discrete Fourier transforms (DFTs), Basic Linear Algebra Subroutines (BLAS) and Linear Algebra PACKage (LAPACK) for linear algebra. Many software, for instance R [7] and Gromacs [8], were configured to support these numerical libraries to improve their performance. It also implements parallel version of several intensive computing programs using Message Passing Interface (MPI), including Gromacs and ClustalW [9].

LXtoo also contains popular scripting languages including Python with BioPython and matplotlib supported, Perl with BioPerl and many third-party tools supported, and R with GO [10], DO [11], KEGG [12], and about 20 whole genome annotation packages [13], our in-house developed packages including GOSemSim [14], DOSE and clusterProfiler [15], and several packages for microarray, proteomics, and FDR analysis [13]. LXtoo features genuine open-source programs, such as NCBI-tools and EMBOSS [16] for sequence analysis, Vienna RNA for RNA secondary structural prediction and comparison, Chimera [17] for molecular assemblies, docking and conformational ensembles, Gromacs [8] for molecular dynamics, Cytoscape [18] and igraph for network analysis, MeV for microarray analysis, and MSnbase [19] for processing MS-based proteomics data, to name a few. LXtoo is trying to integrate software to provide analysis solutions. For instance, microarray data analysis [13] and network modelling tools [18] can work together for investigating regulatory networks differences between normal and diseased cell types; another example by combining miRNA target prediction [20], GO semantic similarity measurement [14], clustering [21] and enrichment analysis [15] for characterizing functional similarity of miRNAs as demonstrated in our previous publication [22]. Shell scripts are under development to supply adaptable and maintainable analysis pipelines.

LXtoo features the Lightweight X11 Desktop Environment (LXDE), which is a fast-performing and energy-saving desktop environment with a clean look and feel [23]. The kernel of LXtoo was configured to use the proper kernel mode setting (KMS) driver for video cards from Intel, nVidia or AMD/ATI; LXtoo can automatically detect hardware and configure X11 windows system at startup. LXtoo had been tested and run fine on 1 GHz CPU with 512 MB RAM, and on DELL Optiplex 960 workstation, which features 2.66 GHz Quad-Core Intel Xeon, 8 GB RAM and ATI Radeon HD 4600 video card. Since most of the bioinformatics analysis is computational intensive, and needs faster processor and more memory, we recommend users running LXtoo with high-performance workstation.

All of the other existing Linux distributions for bioinformatics are binary-based systems. Installing software in these systems will pull in whatever libraries the developer has decided to be included. In contrast, LXtoo is a source-based distribution, users can decide what optimizations are applied and which features will be built into the program when installing software. LXtoo utilizes all the features and advantages of Portage, without losing the Gentoo characteristics. Portage, which is similar to the BSD-style package management, is the default package management system of LXtoo. Portage is completely written in Python and Bash, and thus fully visible to the users, for both are scripting languages. It also benefits solid documentation and strong community as supported from Gentoo community.

LXtoo is entirely based on FOSS software, which is free for redistribution, application and alteration under GPL license. LXtoo is distributed as a Live DVD, and can be booted from the DVD without making any change to the underlying operating system, or run within the popular virtualization software VMware, in parallel with the host operating system. This makes the system portable and useful for new users to try it out, or for those want to demonstrate the software to others. LXtoo can also be booted from Live USB using UNetbootin to create a bootable Live USB [24]. Certainly, installation of LXtoo to hard drive is recommended for advanced users. Runing-LXtoo on hard drive have several advantages, including much better performance since DVD drive is physically slower than hard drive, keeping data and settings persistence after shut-down and flexibility to extend LXtoo. The installation guide is provided in the LXtoo website.

## Conclusions

LXtoo aims to offer biologists with a well-integrated and user-friendly bioinformatics environment in order to alleviate the shortage of bioinformatics expertise. The operating system features a large number of tools that would allow users to run analyses, write scripts, and generally use tools to a high level. LXtoo met all the standards described by Tchantchaleishvili [25], and can served as an ideal platform for creating scientific manuscripts. LXtoo is under active development and undergoes yearly updates to update software, fix bugs, and incorporate new features. Users are encouraged to request additional software or features that would help LXtoo to further evolve to meet the needs of the bioinformatics community. A future version of LXtoo is being developed by providing bioinformatics analysis pipelines based on shell scripts to automate the complex series of analysis steps, and supplying a user-friendly web-based interface to these analysis recipes. The current release of LXtoo is designed for 32-bit architecture and is freely available at http://bioinformatics.jnu.edu.cn/LXtoo. Next release of LXtoo will come in both 32-bit and 64-bit versions.

## Availability and requirements

**Project name**: LXtoo

**Project home page**: http://bioinformatics.jnu.edu.cn/LXtoo/

**Other requirements**: LXtoo runs well on 1 GHz CPU with 512 MB RAM; Quad-Core CPU with 8 GB RAM is good enough for most of the bioinformatics software packages.

## Competing interests

The authors declare that they have no competing interests.

## Authors’ contributions

G Yu and LG Wang developed LXtoo and drafted the manuscript. XH Meng and QY He supervised the study. All authors approved the final manuscript.

## References

[B1] Watson JD The human genome project: past, present, and future Science 1990 248 44 49 2181665 10.1126/science.2181665

[B2] Metagenomics versus Moore’s law Nat Methods 2009 6 623 623 10.1038/nmeth0909-623

[B3] Thomson RC PhyLIS: a simple GNU/Linux distribution for phylogenetics and phyloinformatics Evol Bioinforma 2009 5 91 95 10.4137/ebo.s3169 PMC2747129 19812729

[B4] Field D Tiwari B Booth T Houten S Swan D Bertrand N Thurston M Open software for biologists: from famine to feast Nat Biotechnol 2006 24 801 803 16841067 10.1038/nbt0706-801

[B5] Geeleher P Morris D Hinde JP Golden A BioconductorBuntu: a Linux distribution that implements a web-based DNA microarray analysis server Bioinformatics 2009 25 1438 1439 19307241 10.1093/bioinformatics/btp165

[B6] Bassi S Gonzalez V DNALinux virtual desktop edition Nature Precedings 2007

[B7] Ihaka R Gentleman R R: a language for data analysis and graphics J Comput Graph Stat 1996 5 299 314

[B8] Van Der Spoel D Lindahl E Hess B Groenhof G Mark AE Berendsen HJC GROMACS: fast, flexible, and free J Comput Chem 2005 26 1701 1718 16211538 10.1002/jcc.20291

[B9] Larkin MA Blackshields G Brown NP Chenna R McGettigan PA McWilliam H Valentin F Wallace IM Wilm A Lopez R Thompson JD Gibson TJ Higgins DG Clustal W and Clustal X version 2.0 Bioinformatics 2007 23 2947 2948 17846036 10.1093/bioinformatics/btm404

[B10] Ashburner M Ball CA Blake JA Botstein D Butler H Cherry JM Davis AP Dolinski K Dwight SS Eppig JT Harris MA Hill DP Issel-Tarver L Kasarskis A Lewis S Matese JC Richardson JE Ringwald M Rubin GM Sherlock G Gene ontology: tool for the unification of biology Nat Genet 2000 25 25 29 10802651 10.1038/75556 PMC3037419

[B11] Osborne J Flatow J Holko M Lin S Kibbe W Zhu L Danila M Feng G Chisholm R Annotating the human genome with disease ontology BMC Genomics 2009 10 S6 19594883 10.1186/1471-2164-10-S1-S6 PMC2709267

[B12] Kanehisa M Goto S Furumichi M Tanabe M Hirakawa M KEGG for representation and analysis of molecular networks involving diseases and drugs Nucleic Acids Res 2010 38 D355 D360 19880382 10.1093/nar/gkp896 PMC2808910

[B13] Gentleman RC Carey VJ Bates DM Bolstad B Dettling M Dudoit S Ellis B Gautier L Ge Y Gentry J Hornik K Hothorn T Huber W Iacus S Irizarry R Leisch F Li C Maechler M Rossini AJ Sawitzki G Smith C Smyth G Tierney L Yang JYH Zhang J Bioconductor: open software development for computational biology and bioinformatics Genome Biol 2004 5 R80 15461798 10.1186/gb-2004-5-10-r80 PMC545600

[B14] Yu G Li F Qin Y Bo X Wu Y Wang S GOSemSim: an R package for measuring semantic similarity among GO terms and gene products Bioinformatics 2010 26 976 978 20179076 10.1093/bioinformatics/btq064

[B15] Yu G Wang L-G Han Y He Q-Y clusterProfiler: an R package for comparing biological themes among gene clusters OMICS: J Integr Biol 2012 16 284 287 10.1089/omi.2011.0118 PMC3339379 22455463

[B16] Rice P Longden I Bleasby A EMBOSS: the European Molecular Biology Open Software Suite Trends Genet 2000 16 276 277 10827456 10.1016/s0168-9525(00)02024-2

[B17] Pettersen EF Goddard TD Huang CC Couch GS Greenblatt DM Meng EC Ferrin TE UCSF Chimera–a visualization system for exploratory research and analysis J Comput Chem 2004 25 1605 1612 15264254 10.1002/jcc.20084

[B18] Shannon P Markiel A Ozier O Baliga NS Wang JT Ramage D Amin N Schwikowski B Ideker T Cytoscape: a software environment for integrated models of biomolecular interaction networks Genome Res 2003 13 2498 2504 14597658 10.1101/gr.1239303 PMC403769

[B19] Gatto L Lilley KS MSnbase – an R/Bioconductor package for isobaric tagged mass spectrometry data visualization, Processing and quantitation Bioinformatics 2012 28 288 289 22113085 10.1093/bioinformatics/btr645

[B20] Kertesz M Iovino N Unnerstall U Gaul U Segal E The role of site accessibility in microRNA target recognition Nat Genet 2007 39 1278 1284 17893677 10.1038/ng2135

[B21] Suzuki R Shimodaira H Pvclust: an R package for assessing the uncertainty in hierarchical clustering Bioinformatics 2006 22 1542 1540 10.1093/bioinformatics/btl117 16595560

[B22] Yu G He Q-Y Functional similarity analysis of human virus-encoded miRNAs J Clin Bioinforma 2011 1 15 21884632 10.1186/2043-9113-1-15 PMC3164608

[B23] Powers S The second-string desktop Linux J 2011 202 2

[B24] Elmendorf D Economy size geek: installation toolkit Linux J 2010 191 10

[B25] Tchantchaleishvili V Schmitto JD Preparing a scientific manuscript in Linux: today’s possibilities and limitations BMC Res Notes 2011 4 434 22018246 10.1186/1756-0500-4-434 PMC3227619

